# Knockdown of lncRNA MALAT1 ameliorates acute kidney injury by mediating the miR‐204/APOL1 pathway

**DOI:** 10.1002/jcla.23881

**Published:** 2021-07-09

**Authors:** Hai‐Yuan Lu, Guo‐Yi Wang, Jin‐Wen Zhao, Hai‐Tao Jiang

**Affiliations:** ^1^ Department of Nephrology The Affiliated Huaian No.1 People’s Hospital of Nanjing Medical University Huai’an China; ^2^ Department of Orthopedics Huai’an First People’s Hospital Huai’an China

**Keywords:** acute kidney injury, APOL1, inflammation, lncRNA MALAT1, miR‐204

## Abstract

**Background:**

Acute kidney injury (AKI) was characterized by loss of renal function, associated with chronic kidney disease, end‐stage renal disease, and length of hospital stay. Long non‐coding RNAs (lncRNAs) participated in AKI development and progression. Here, we aimed to investigate the roles and mechanisms of lncRNA MALAT1 in AKI.

**Methods:**

AKI serum samples were obtained from 129 AKI patients. ROC analysis was conducted to confirm the diagnostic value of MALAT1 in differentiating AKI from healthy volunteers. After hypoxic treatment on HK‐2 cells, the expressions of inflammatory cytokines, MALAT1, miR‐204, APOL1, p65, and p‐p65, were measured by RT‐qPCR and Western blot assays. The targeted relationship between miR‐204 and MALAT1 or miR‐204 and APOL1 was determined by luciferase reporter assay and RNA pull‐down analysis. After transfection, CCK‐8, flow cytometry, and TUNEL staining assays were performed to evaluate the effects of MALAT1 and miR‐204 on AKI progression.

**Results:**

From the results, lncRNA MALAT1 was strongly elevated in serum samples from AKI patients, with the high sensitivity and specificity concerning differentiating AKI patients from healthy controls. In vitro, we established the AKI cell model after hypoxic treatment. After experiencing hypoxia, we found significantly increased MALAT1, IL‐1β, IL‐6, and TNF‐α expressions along with decreased miR‐204 level. Moreover, the targeted relationship between MALAT1 and miR‐204 was confirmed. Silencing of MALAT1 could reverse hypoxia‐triggered promotion of HK‐2 cell apoptosis. Meanwhile, the increase of IL‐1β, IL‐6, and TNF‐α after hypoxia treatment could be repressed by MALAT1 knockdown as well. After co‐transfection with MALAT1 silencing and miR‐204 inhibition, we found that miR‐204 could counteract the effects of MALAT1 on HK‐2 cell progression and inflammation after under hypoxic conditions. Finally, NF‐κB signaling was inactivated while APOL1 expression was increased in HK‐2 cells after hypoxia treatment, and lncRNA MALAT1 inhibition reactivated NF‐κB signaling while suppressed APOL1 expression by sponging miR‐204.

**Conclusions:**

Collectively, these results illustrated that knockdown of lncRNA MALAT1 could ameliorate AKI progression and inflammation by targeting miR‐204 through APOL1/NF‐κB signaling.

## Graphical abstract

lncRNA MALAT1 was strongly elevated in serum samples from AKI patients, with the high sensitivity and specificity concerning differentiating AKI patients from healthy controls. Silencing of MALAT1 could reverse hypoxia‐triggered promotion of HK‐2 cell apoptosis. miR‐204 could counteract the effects of MALAT1 on HK‐2 cell progression and inflammation after under hypoxic conditions. NF‐κB signaling was inactivated while APOL1 expression was increased in HK‐2 cells after hypoxia treatment, and lncRNA MALAT1 inhibition reactivated NF‐κB signaling while suppressed APOL1 expression by sponging miR‐204.

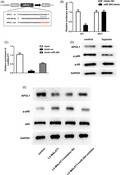

## 1 INTRODUCTION

Sepsis was caused by a fatal systemic inflammatory syndrome that can lead to shock, multiple organ dysfunction syndromes, and even death.^1^ Globally, the incidence of sepsis increased dramatically, with more than 30 million patients and nearly 6 million deaths each year.^2^ Sepsis may be caused by a variety of infections and other non‐infectious diseases, such as ischemia, trauma, drug reactions, or even cancer, but the underlying pathogenetic mechanisms remained unclear.^3^ Acute kidney injury (AKI), a severe syndrome associated with renal insufficiency, was one of the most serious complications of sepsis.^3^ It can trigger the development of organ dysfunction, which led to high morbidity and mortality in most patients with sepsis.^4^ Studies found that severe sepsis can result in approximately 50% of AKI cases.^5^ Therefore, exploring the mechanisms of pathophysiological alterations of sepsis‐induced AKI patients was a challenging and necessary task.

There was strong evidence that renal tubular epithelial cell injury, inflammation, vascular dysfunction, and fibrosis were involved in the pathology and physiology of AKI.^6^ Sepsis promoted the release of inflammatory factors from renal tissues, resulting in apoptosis of renal cells, which led to AKI.^7^ Therefore, improving the level of inflammation and apoptosis in patients with a renal injury would be of great benefit to patients with AKI. Despite advances in therapeutic approaches, the mortality rate of sepsis‐induced AKI remained high. Therefore, it is necessary and urgent to explore new therapeutic targets to improve the survival of patients with septic AKI.

Long non‐coding RNAs (lncRNAs), located in the nucleus or cytoplasm, were a class of ncRNAs with more than 200 nucleotides.^8^ LncRNAs have been verified to exert significant roles in biological processes such as cell differentiation, cell proliferation, apoptosis, and tumorigenesis.^9^, ^10^ Recent evidence suggested that lncRNAs may also be involved in the pathophysiology of AKI. For instance, downregulation of LncRNA MEG3 may protect against AKI induced by hypoxia/reoxygenation in HK‐2 cells by regulating the miR‐129‐5p/MHGB1 axis.^11^ LINC00520 may promote the development of AKI by regulating miR‐27b‐3p/OSMR/PI3K/AKT signaling pathway.^12^ Silencing of lncRNA NEAT1 can target miR‐125‐5p and regulate TRAF6/TAK1 signaling to protect against sepsis‐induced AKI.^13^ LncRNA MALAT1 exerted either oncogenetic or tumor‐suppressive roles in various cancers, including lung, breast, gastric, gallbladder, and colorectal cancers.^14^, ^15^, ^16^, ^17^, ^18^ In AKI, lncRNA MALAT1 was proved to be upregulated in AKI patients.^19^ However, the role of lncRNA MALAT1 in the pathology and physiology of sepsis still needed to be further explored.

In this study, we investigated the expression of lncRNA MALAT1 in the serums of AKI patients and its clinical diagnostic value. Hypoxia/reoxygenation‐treated HK‐2 cells were applied to establish in vitro AKI cell model. In addition, we investigated whether MALAT1 could regulate cellular progression and inflammatory response by regulating the miR‐204/APOL1/NF‐κB axis in AKI. Collectively, this study may help to explore new therapeutic approaches for AKI caused by sepsis.

## 2 METHODS

### 2.1 Specimen collection

A 129 AKI patients and 100 healthy volunteers were recruited from Huai'an First People's Hospital between December 2015 and April 2019 (Table 1). The clinical diagnostic guidelines developed by the Kidney Disease Prognosis Improvement Group (KDIGO) in 2012 define AKI as a sudden decline in renal function within 48 h, with an increase in absolute serum creatinine ≥ 0.3 mg/dl (26.5 μmol/L), or an increase in serum creatinine to more than 1.5 times than the basal value within 7 days, or a urine output ≤0.5 m/(kg/h) for >6 h. The inclusion criteria for AKI patients were as follows: (1) patients who met the AKI diagnostic criteria of KDIGO in 2012; (2) age >18 years; (3) admission time more than 48 h; and (4) patients with complete medical history and signed informed consent. Meanwhile, according to KDIGO 2012, all AKI patients were classified as stage 1, stage 2, and stage 3 groups. Serum samples were extracted from all subjects and centrifugated at 5000 *g* for 3 min at 4℃. The present study was approved by the Ethics Committee of Huai'an First People's Hospital following the Helsinki Declaration. Written informed consent was obtained from each participant.

**TABLE 1:** Clinicopathological information of AKI and non‐AKI healthy patients

Parameters	Healthy (*N* = 100)	AKI (*N* = 129)	*p* value
Age (years)	52.45 ± 8.12	51.73 ± 11.01	0.5840
Gender			
Male	67	75	0.1706
Female	33	54	
BMI (kg/m^2^)	20.98 ± 1.57	21.06 ± 1.98	0.7408
Hypertension history	9	11	0.8999
Diabetes history	6	8	0.9497
Cardiovascular diseases history	7	8	0.8086
CRP (ng/ml)	65.08 ± 21.97	83.79 ± 31.04	<0.001
eGFR (ml/min/1.73m^2^)	62.07 ± 19.02	52.67 ± 22.73	<0.001
Scr (μM)	99.65 ± 24.71	156.28 ± 26.75	<0.001
Cys‐C (mg/L)	0.58 ± 0.17	1.98 ± 0.60	<0.001
NGAL (ng/ml)	55.69 ± 17.32	81.57 ± 19.68	<0.001
KIM−1 (ng/ml)	4.86 ± 0.58	23.91 ± 6.59	<0.001
MALAT1 level (fold)	1.02 ± 0.32	1.66 ± 0.21	<0.001
miR−204 level (fold)	0.99 ± 0.23	0.34 ± 0.11	<0.001

Abbreviations: BMI, body mass index; CRP, c‐reaction protein; Cys‐C, cystatin‐C; eGFR, estimated glomerular filtration; KIM‐1, kidney injury molecule‐1; NGAL, neutrophil gelatinase‐associated lipocalin; Scr, serum creatinine.

The work described in our article was carried out in accordance with the Code of Ethics of the World Medical Association (Declaration of Helsinki).

### 2.2 Renal function assessment

Demographic and clinical parameters of all subjects were recorded in Table 1. After centrifugation, serum creatinine (SCr) was measured by corresponding detection kits (Nanjing Jian Cheng Institute of Biotechnology, Nanjing, China).

### 2.3 Cell culture and hypoxic treatment

HK‐2 and HEK‐293T cells were purchased from Cell Bank of Type Culture Collection of the Chinese Academy of Sciences (Shanghai, China) and cultured in RPMI‐1640 medium (Gibco, USA) supplemented with penicillin‐streptomycin and 10% fetal bovine serum (FBS; Gibco, USA) in a humidified incubator with 5% CO_2_, at 37℃.

To establish the AKI cell model, HK‐2 cells were exposed to hypoxia (94% N_2_, 5% CO_2_, 1% O_2_) for 24 h followed by 12 h of reoxygenation (74% N_2_, 5% CO_2_, 21% O_2_) at 37℃.

### 2.4 Lentiviral infection and transfection

LV‐NC, LV‐MALAT1, miR‐204 inhibitor, inhibitor‐NC, mimic‐NC, miR‐204 mimic, si‐NC, and si‐APOL1 were designed, constructed, and purchased from GenePharma (Shanghai, China). Briefly, LV‐MALAT was sub‐cloned into lentiviral plasmids to infect HEK‐293T cells along with lentiviral packaging plasmids. Afterward, cell transfection was conducted using Lipofectamine 2000 (Thermo Fisher Scientific, Inc, Shanghai, China) as per the manufacturers’ instructions.

### 2.5 RT‐qPCR analysis

Total RNAs were isolated from serum samples and cells using TRIzol reagent (Invitrogen; Thermo Fisher Scientific, Inc., Shanghai, China) as per the instructions. Then, cDNA was reverse synthesized from RNAs using a reverse transcript kit (Takara, Japan). RT‐q PCR was then conducted via SYBR Green PCR Master Mix (Takara, Japan) on a 7500 ABI real‐time PCR instrument (Applied Biosystems; Thermo Fisher Scientific, Inc., Shanghai, China). The expressions of lncRNA MALAT1 and miR‐240 were normalized to that of U6, calculated with the 2^−ΔΔCT^ method. The primers sequences were as follows: IL‐1β (forward), 5′‐ATGATGGCTTATTACAGTGGCAA‐3′, and (reverse) 5′‐GTCGGAGATTCGTAGCTGGA‐3′; IL‐6 (forward), 5′‐ACTCACCTCTTCAGAACGAATTG‐3′, and (reverse) 5′‐CCATCTTTGGAAGGTTCAGGTTG‐3′; TNF‐α (forward), 5′‐CTCTTCTGC CTGCTGCACTTTG‐3′, and (reverse) 5′‐ATGGGCTACAGGCTTGTCACTC‐3′; lncRNA MALAT1 (forward), 5′‐TGTGACGCGACTGGAGTATG‐3′, and (reverse) 5′‐CAAAGGGACTCGGCTCCAAT‐3′; miR‐204 (forward), 5′‐TGGCTACAGTCTTTCTTCA‐3′, and (reverse) 5′‐CTCATGGGACAGTTATGG‐3′; APOL1 (forward), 5′‐TAAGGTACCGACAGAGGGAGGCAGCC‐3′, and (reverse) 5′‐ACCGTCGACTCAGAAGGGTGCCAGACCC‐3′; U6 (forward), 5′‐GCTTCGGCAGCACATATACTAAAAT‐3′, and (reverse) 5′‐CGCTTCACGAATTTGCGTGTCAT‐3′; GAPHD (forward), 5′‐TGCACCACCAACTGCTTAGC‐3′, and (reverse) 5′‐GGCATGGACTGTGGTCATGAG‐3′.

### 2.6 Dual‐luciferase reporter assay

The lncRNA MALTA1 3’UTR and APOL1 3’UTR were cloned into the pGL3‐Basic vector, while mutant MALAT1 3’UTR and APOL1 3’UTR were cloned into the pGL3 luciferase vector. Then, HK‐2 cells were transfected with APOL1 3’UTR, mutant APOL1 3’UTR, MALAT1 3’UTR, mutant MALAT1 3’UTR, followed transfected with miR‐204 mimic or mimic‐NC using Lipofectamine 2000 (Thermo Fisher Scientific, Inc., Shanghai, China) following the protocol. Relative luciferase activity was normalized to *Renilla* activity on a dual‐luciferase reporter assay system (Promega) according to the manufacturer's protocol.

### 2.7 RNA pull‐down assay

HK‐2 cells were transfected with biotinylated miR‐204, biotinylated mutant miR‐204, or biotinylated negative controls (GenePharma, Shanghai, China). Then, lysates were incubated with M‐280 streptavidin magnetic beads. Finally, RT‐qPCR was conducted to measure the bound RNAs.

### 2.8 CCK‐8 assay

After 24 h of transfection, treated cells were inoculated in a 6‐well plate at the density of 1×10^5^ cells/well, cultivated in a humidified incubator with 5% CO_2_ at room temperature. At 0, 24, and 48 h, 10 μl CCK‐8 reagent (Dojindo, Japan) was added to each well and cultured for another 2 h. Finally, a microplate reader (Dynatech, VA) was used to detect the absorbance value at 450 nm.

### 2.9 Colony formation analysis

Transfected cells of logarithmic growth phase were taken, digested with 0.25% trypsin and blown into individual cells, and the cells were suspended in RPMI‐1640 medium with 10% FBS. The cell suspension was diluted in a gradient multiple, and each group of cells was inoculated in a culture dish containing 10 ml of 37°C pre‐warmed culture medium. The cells were incubated for 2 weeks at 37°C with 5% CO_2_ and saturated humidity in a cell incubator. 5 ml of 4% paraformaldehyde was added to fix cells for 15 min. Finally, GIMSA staining solution was added for 30 min.

### 2.10 Flow cytometry analysis

After transfection, cells were plated in 96‐well plates with a density of 2×10^4^ cells/well at 37℃, 5% CO_2_. After incubation for 48 h, cells were stained with Annexin V/FITC and PI reagent (BD, USA) at room temperature in the dark for 20 min. The apoptotic cells were measured by FACS Calibur flow cytometer (BD Biosciences, USA) as per the protocol.

### 2.11 TUNEL staining analysis

Treated cell smears were naturally dried and fixed at room temperature for 30 min using 4% paraformaldehyde. After washing with PBS three times, samples were immersed in the sealing solution (3% H_2_O_2_, methyl) and blocked at room temperature for 10min. Then, samples were immersed in cell membrane permeabilization solution (0.1% Triton X‐100) for 2 min at 37℃. Next, samples were stained with TUNEL Apoptosis Detection Kit (Roche, Germany) and counterstained with DAPI. Finally, a fluorescence microscope system (Carl Zeiss, Germany) was utilized to capture the images.

### 2.12 Western blot assay

Total proteins were extracted using RIPA lysis buffer (Sigma, USA) and quantified with a BCA detecting kit (Abcam, Shanghai, China) following the manufacturers’ instructions. Then, proteins were separated by 10% SDS‐PAGE and transferred on a PVDF membrane (BMD Millipore, Billerica, USA). After blocking with 5% skimmed milk for 2 h in the dark at 37℃, membranes were incubated with rabbit anti‐APOL1 (ab108315; Abcam, Shanghai, China), rabbit anti‐p65 (ab16502; Abcam, Shanghai, China), rabbit anti‐p‐p65 (ab76302; Abcam, Shanghai, China), and rabbit anti‐GAPDH (ab9485; Abcam, Shanghai, China) overnight at 4℃. The following day, membranes were then incubated with secondary antibody goat anti‐rabbit IgG H&L (HRP; ab7090; Abcam, Shanghai, China) at room temperature for 50 min. Finally, the blots were visualized with an enhanced chemiluminescence detection system (Pierce, USA) and analyzed with Image Lab Software (Bio‐Rad).

### 2.13 Statistical analysis

All the data in our study were presented as mean ± standard deviation (SD). Student's *t* test and ANOVA tests were used to distinguish differences between groups. ROC analysis was utilized to assess the diagnostic value. SPSS 22.0 (SPSS Inc, Chicago, IL, USA) and Prism 6.0 (GraphPad Software Inc.) were applied to analyze the data. A *p*‐value < 0.05 was considered statistically significant. All experiments were conducted at least in triplicate.

## 3 RESULTS

### 3.1 Clinicopathological information of AKI patients

As shown in Table 1, we found that there were no significant differences among age, gender, body mass index (BMI), hypertension history, diabetes history, and cardiovascular diseases history, whereas AKI was significant associated with c‐reaction protein (CRP), estimated glomerular filtration (eGFR), serum creatinine (Scr), cystatin‐C (Cys‐C), neutrophil gelatinase‐associated lipocalin (NGAL), kidney injury molecule‐1 (KIM‐1), lncRNA MALAT1 level, and miR‐204 level.

### 3.2 LncRNA MALAT1 expression was increased while miR‐204 was diminished in AKI patients and HK‐2 cells after hypoxia treatment

**FIGURE 1:**
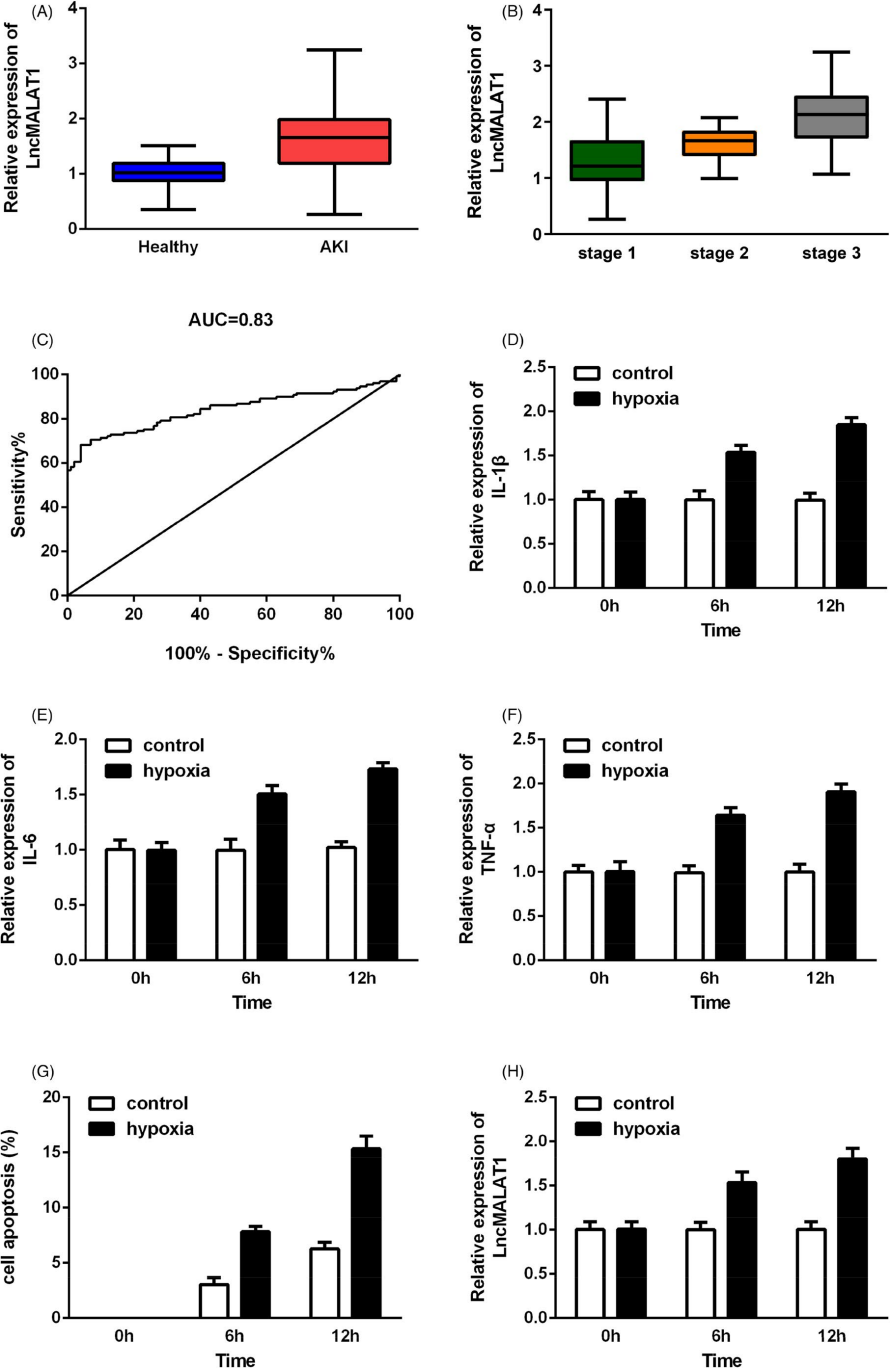
LncRNA MALAT1 was significantly increased in AKI patients and HK‐2 cells after hypoxia treatment. (A) Analysis of MALAT1 expressions in serum samples from AKI patients and healthy controls. (B) Expressions of MALAT1 in AKI patients according to different stages. (C) The diagnostic value of MALAT1 in discriminating AKI patients from healthy controls. (D‐F) IL‐1β, IL‐6, and TNF‐α expressions in HK‐2 cells exposed to hypoxia. (G) The ratio of apoptotic cells in HK‐2 after 0, 6, and 12 h hypoxia. (H) Expressions of MALAT1 in HK‐2 cells before and after hypoxia

**FIGURE 2:**
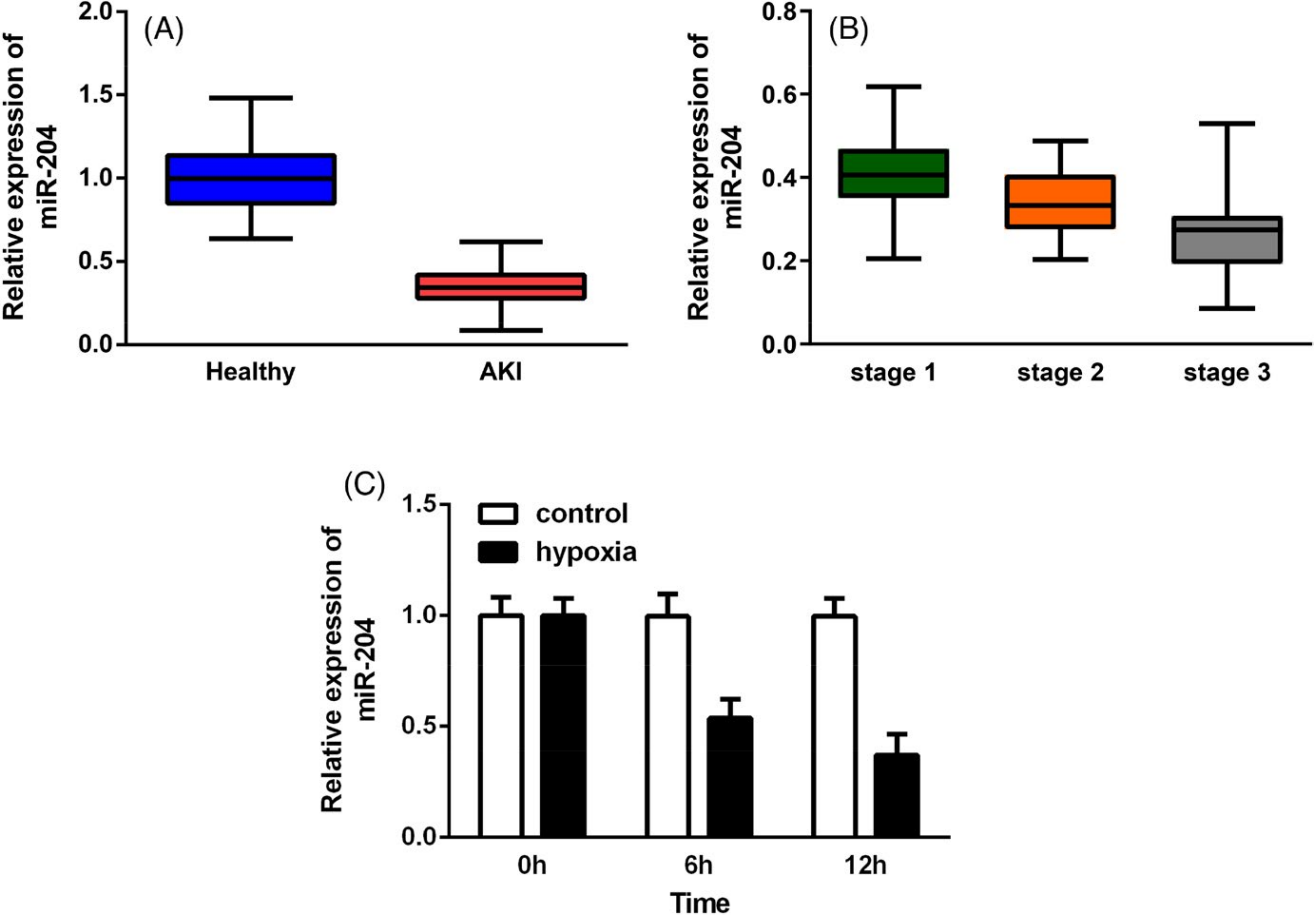
miR‐204 was prominently decreased in AKI patients and HK‐2 cells under hypoxic conditions. (A) Analysis of miR‐204 expressions in AKI patients. (B) Expressions of miR‐204 in AKI patients according to different stages. (C) miR‐204 level in hypoxia‐triggered HK‐2 cells

First, we determined the expressions of lncRNA MALAT1 and miR‐204 in serum samples from AKI patients and healthy controls via RT‐qPCR assays. As shown in Figures 1A and 2A, the level of MALAT1 was elevated while miR‐204 was reduced in AKI patients. Moreover, according to the KDIGO guideline, we found that the increase in MALAT1 or the decrease in miR‐204 was more evident in stage 3 AKI patients compared with stage 1 or stage 2 groups (Figures 1B and 2B). Meanwhile, the ROC analysis in Figure 1C further verified the potential of MALAT1 in discriminating AKI from healthy controls with the specificity of, the sensitivity of, and cutoff value of 0.315 (AUC = 0.8394, 95% CI = 0.7866–0.8921). Next, we developed an AKI cell model by exposing HK‐2 cells to hypoxia. After 12 h exposure to hypoxia, we found that IL‐1β, IL‐6, and TNF‐α levels were increased in a time‐dependent manner (Figure 1D–F), so as the apoptotic rate in HK‐2 cells (Figure 1G). We then uncovered that MALAT1 expression was upregulated while miR‐204 was diminished after hypoxia treatment (Figures 1H and 2C).

### 3.3 miR‐204 targeted MALAT1

**FIGURE 3:**
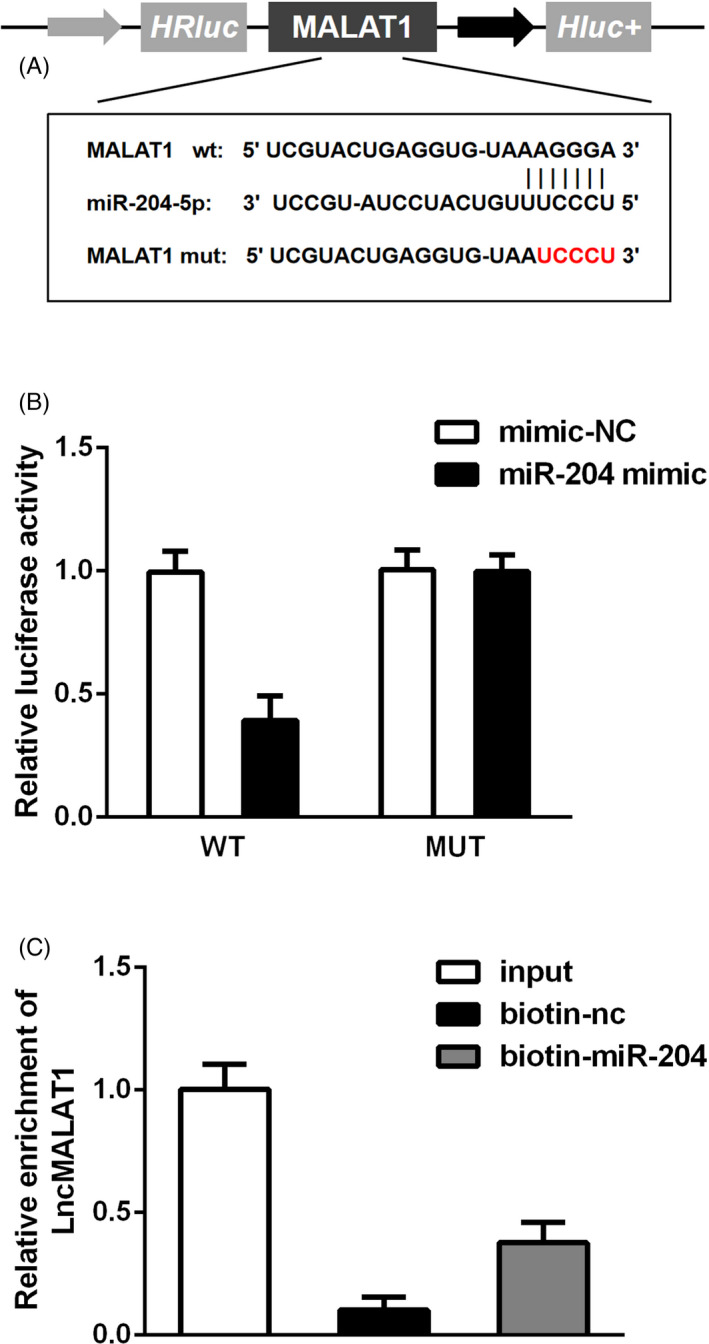
miR‐204 was a target of lncRNA MALAT1. (A) The potential targeted sequences between MALAT1 and miR‐204. (B) Luciferase reporter assay between MALAT1 and miR‐204. (C) RNA pull‐down assay detected the interaction between miR‐204 and MALAT1

The interaction between MALAT1 and miR‐204 was displayed in Figure 3A. The luciferase reporter assay disclosed that luciferase activity in HK‐2 cells after co‐transfection with miR‐204 mimic and MALAT1‐WT was strongly reduced (Figure 3B), whereas there were no significant differences after transfection with miR‐204 mimic and mutant MALAT1. RNA pull‐down analysis in Figure 3C showed a prominently higher MALAT1 while using miR‐204‐bio probes than the NC‐bio or miR‐204 probes.

### 3.4 The effects of MALAT1 on HK‐2 cell progression and inflammation under hypoxic conditions were partially reversed by co‐transfection with miR‐204 inhibitor

**FIGURE 4:**
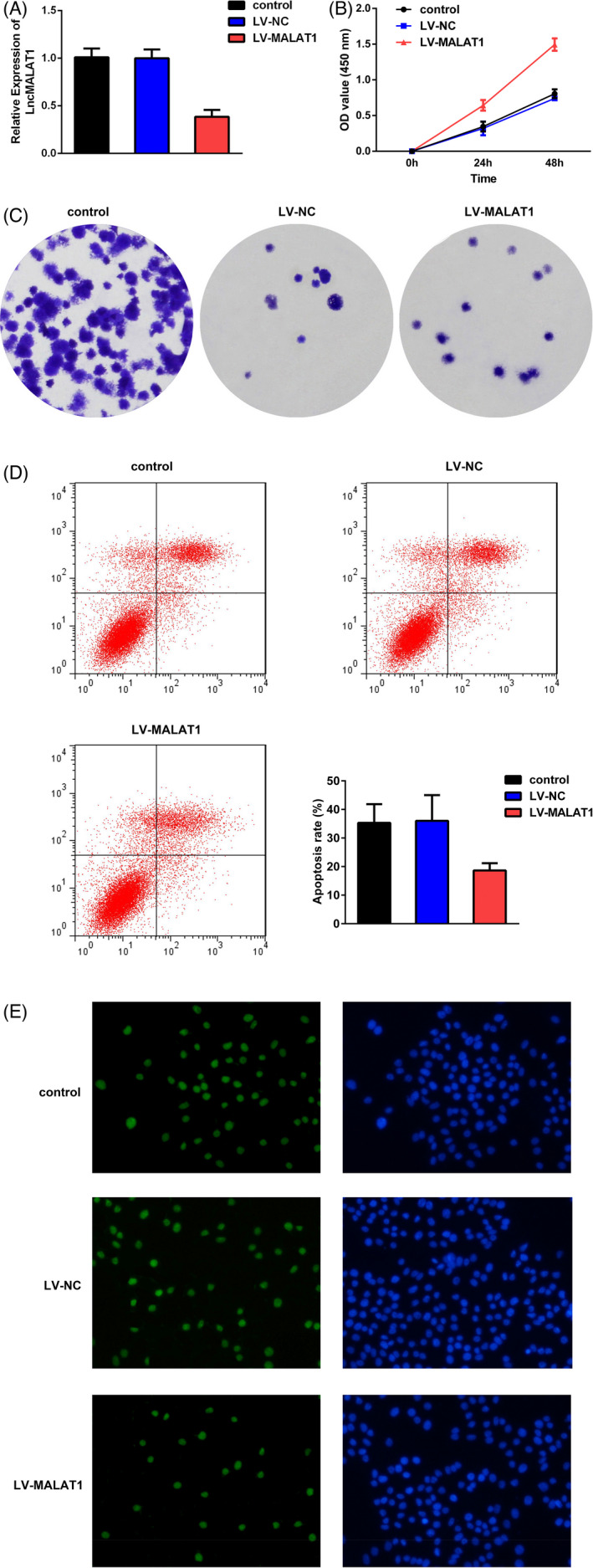
Silencing of MALAT1 inhibited HK‐2 cell apoptosis and promoted proliferation after hypoxia. (A) Expression of MALAT1 after infected with LV‐MALAT1 and LV‐NC. (B) CCK‐8 assay displayed cell viability. (C) Colony formation assay displayed cell proliferation. (D) Flow cytometry assays presented the cell apoptosis. (E) Hypoxia‐triggered HK‐2 cell apoptosis after transfection with LV‐MALAT1 was confirmed by TUNEL staining assay

**FIGURE 5:**
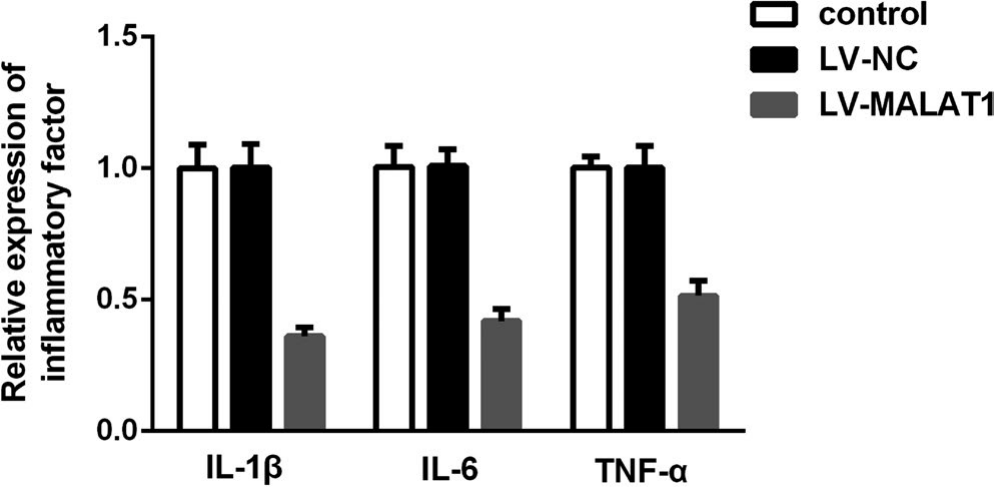
Knockdown of MALAT1 ameliorated inflammation in AKI

To illustrate the effects of MALAT1 on the hypoxia‐triggered AKI cell model, HK‐2 cells were infected with LV‐MALAT1 or LV‐NC. As shown in Figure 4A, MALAT1 expression was found to be markedly decreased in LV‐MALAT1, suggesting the transfection was successful. In addition, as evidenced by the CCK‐8 and colony formation assays, LV‐MALAT1 could promote HK‐2 cell proliferation after hypoxia treatment (Figure 4B,C). Meanwhile, the inflammation in HK‐2 cells induced by hypoxia could be partially ameliorated after knocking down MALAT1 expression (Figure 5). As depicted in Figure 4D,E, flow cytometry and TUNEL staining assays elucidated that hypoxia‐engendered apoptosis in HK‐2 cells could be rescued by transfection with LV‐MALAT1 as well.

### 3.5 miR‐204 targeted APOL1 to abolish the suppressive effects of MALAT1 silencing on HK‐2 cells experiencing hypoxia by activation NF‐κB

**FIGURE 6:**
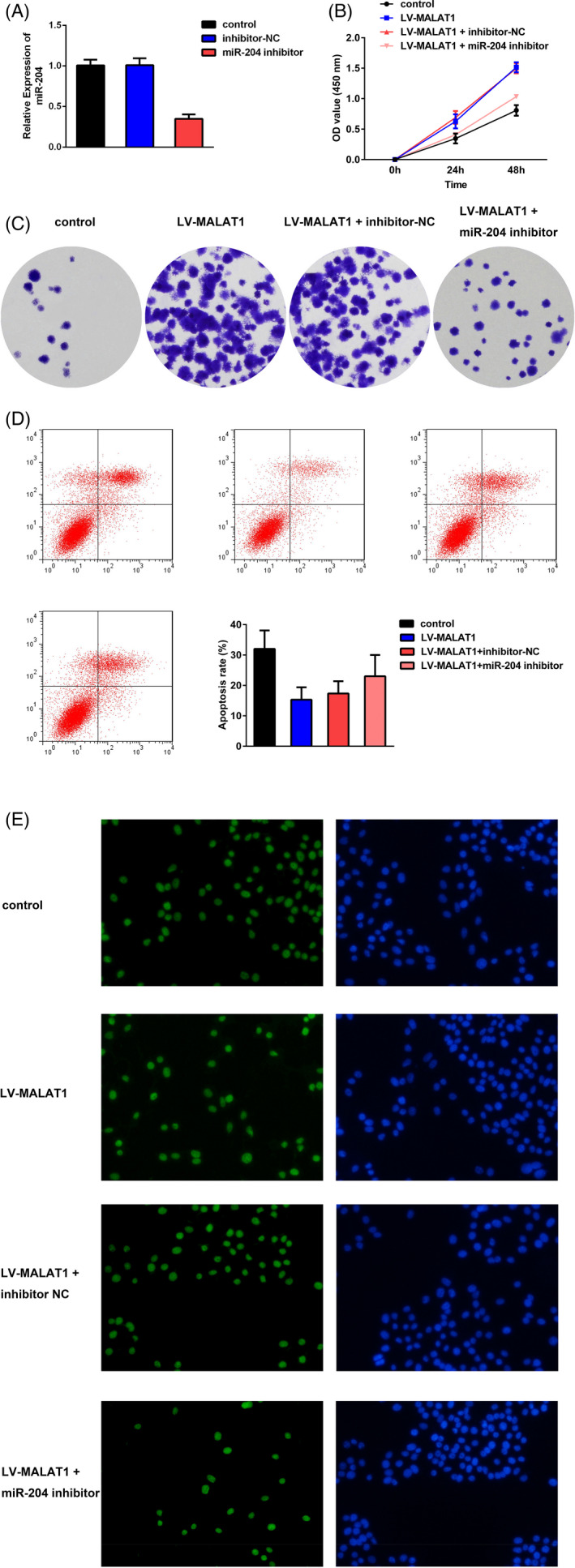
miR‐204 abolished the repressive effects of MALAT1 silencing on HK‐2 cells experiencing hypoxia. (A) Expression of miR‐204 after co‐transfection with miR‐204 inhibitor, inhibitor‐NC, and LV‐MALAT1. (B) CCK‐8 assay. (C) Colony formation results. (D) Flow cytometry assay. (E) TUNEL staining assay

**FIGURE 7:**
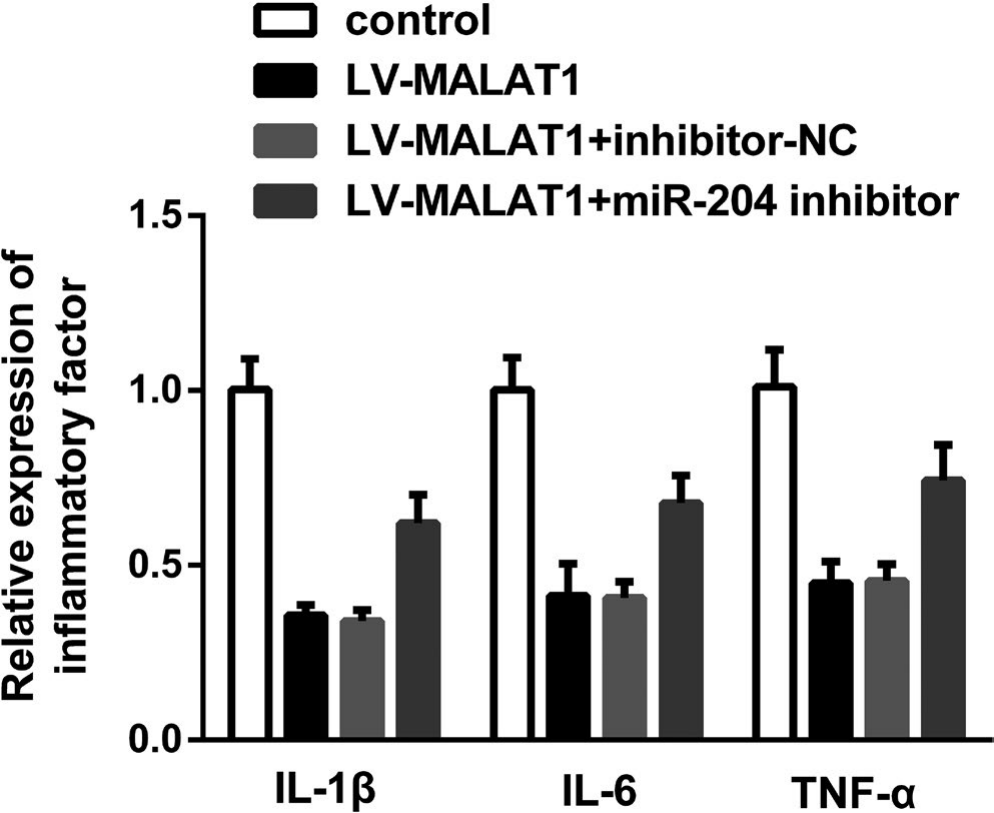
miR‐204 counteracted the impacts of MALAT1 on inflammation in hypoxia‐treated HK‐2 cells

Under hypoxic conditions, HK‐2 cells were co‐transfected with LV‐MALAT1 and miR‐204 inhibitor or inhibitor‐NC. As demonstrated in Figure 6A, the expression of miR‐204 was increased in the LV‐MALAT1 group, while the increase was further hindered after co‐transfection with miR‐204 inhibitor. The CCK‐8, colony formation, flow cytometry, and TUNEL staining assays in Figure 6B‐E illustrated that the inhibition of apoptosis and promotion of proliferation in HK‐2 cells under hypoxia after silencing MALAT1 could be partially reversed by co‐transfected with miR‐204 inhibitor. The inflammation repressed by MALAT1 knockdown was aggravated by transfection with the miR‐204 inhibitor as well (Figure 7).

**FIGURE 8:**
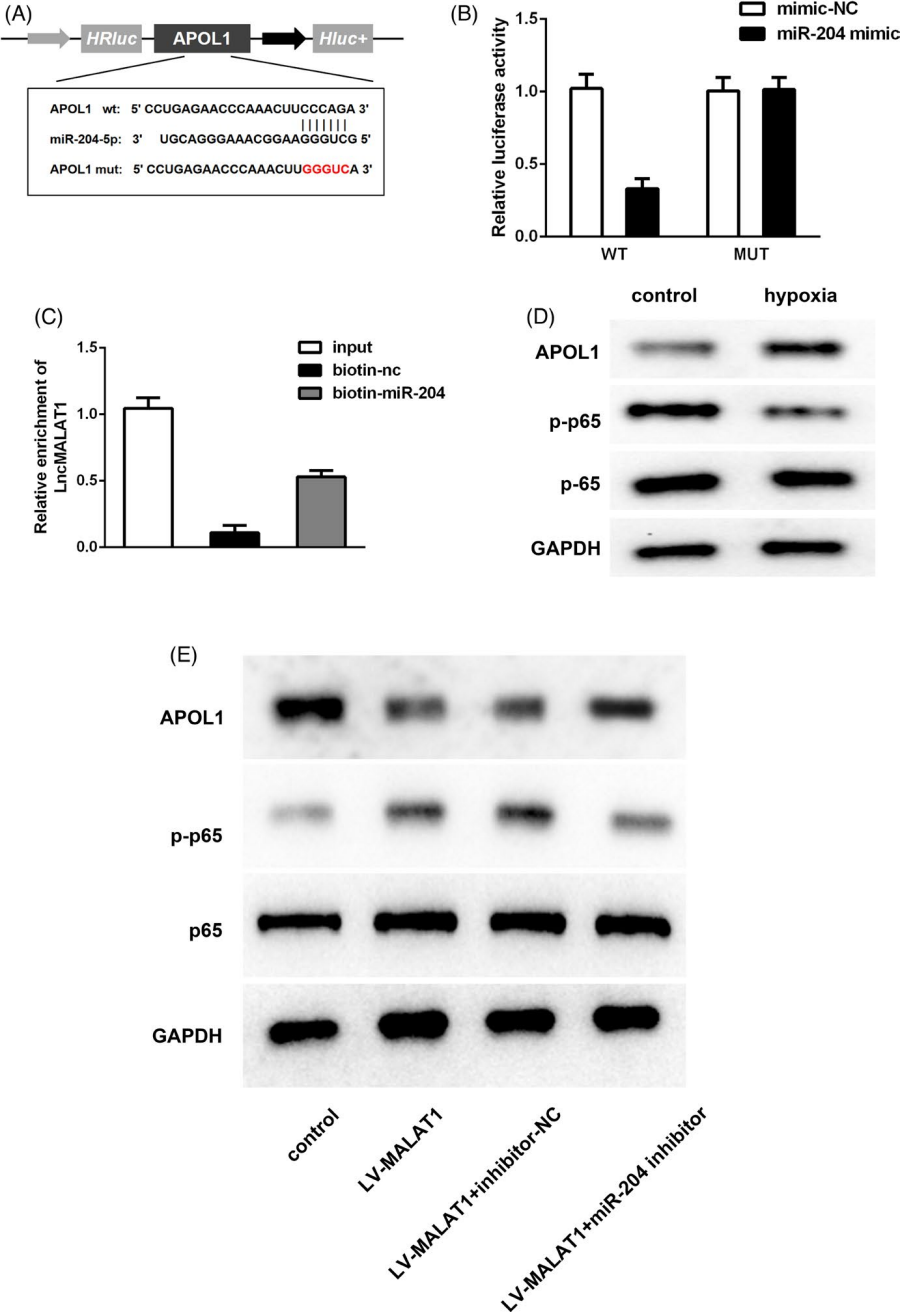
miR‐204 targeted APOL1 to abrogate the inhibitory effects of MALAT1 knockdown by activation of the NF‐κB pathway in HK‐2 cells. (A) The potential binding sequences between APOL1 and miR‐204. (B) Luciferase reporter assay. (C) RNA pull‐down assay. (D) Protein expressions of APOL1, p65, and p65 in HK‐2 cells after hypoxia. (E) APOL1, p65, and p‐p65 levels after co‐transfection with LV‐MALAT1, inhibitor‐NC, and miR‐204 inhibitor in HK‐2 cells exposed to hypoxia

Next, the loop relationship between MALAT1, miR‐204, and APOL1 was confirmed. As evidenced in Figure 8A‐C, we verified the targeted relationship between miR‐204 and APOL1 by luciferase reporter assay and RNA pull‐down analysis. Furthermore, Western blot results in Figure 8D unveiled that APOL1 expression was increased while p‐p65 was decreased after hypoxia treatment in HK‐2 cells. Meanwhile, under hypoxic conditions, APOL1 was reduced while p‐p65 was elevated after transfection with LV‐MALAT1; however, the variance induced by LV‐MALAT1 could be partially rescued by a miR‐204 inhibitor (Figure 8E).

**FIGURE 9:**
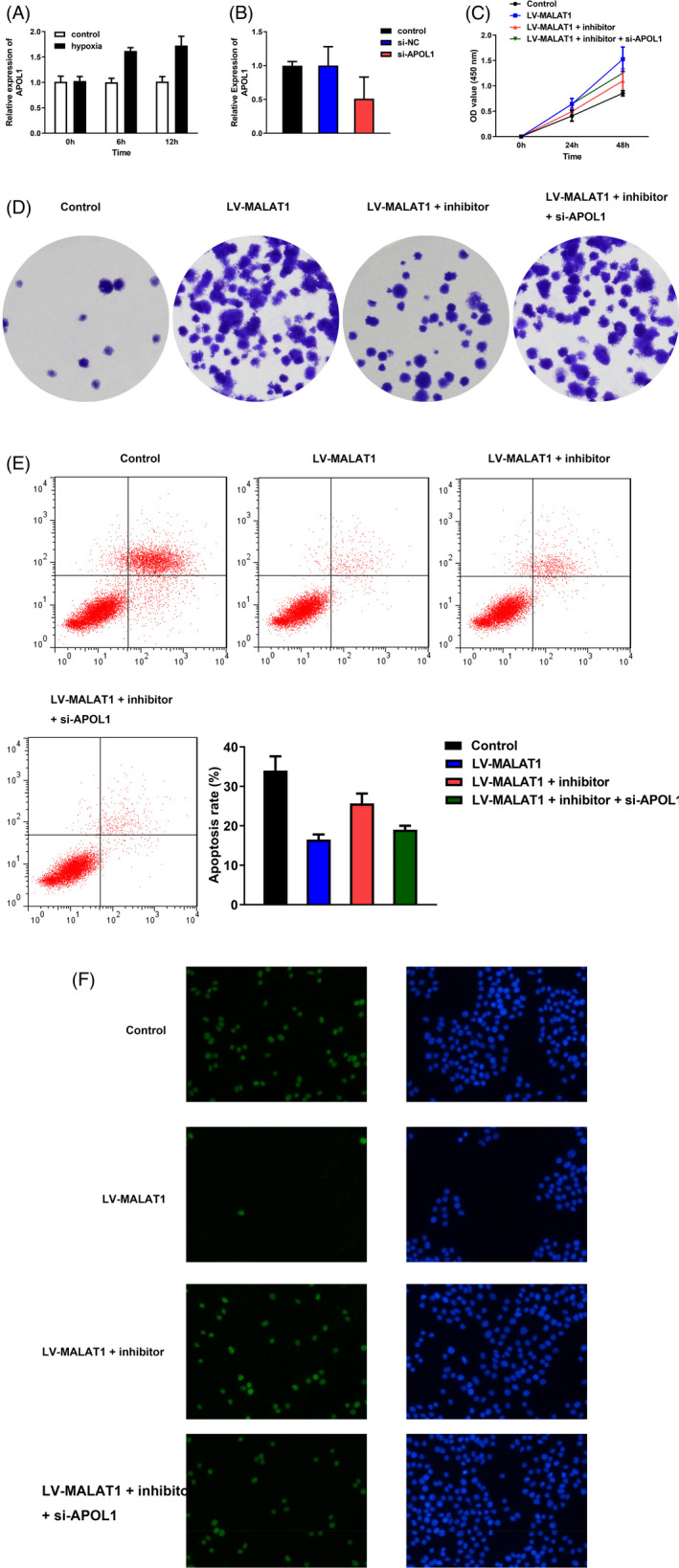
Effects of MALAT1/miR‐204/APOL1 axis on HK‐2 cell proliferation and apoptosis under hypoxic conditions. (A) Expression of APOL1 after 12‐h hypoxia treatment in HK‐2 cells. (B) Transfection efficacy of APOL1 in hypoxia‐induced HE‐2 cells. (C) CCK‐8 results. (D) Cell viability evaluated by colony formation analysis. (E) Cell apoptosis determined by flow cytometry analysis. (F) TUNEL staining assay

**FIGURE 10:**
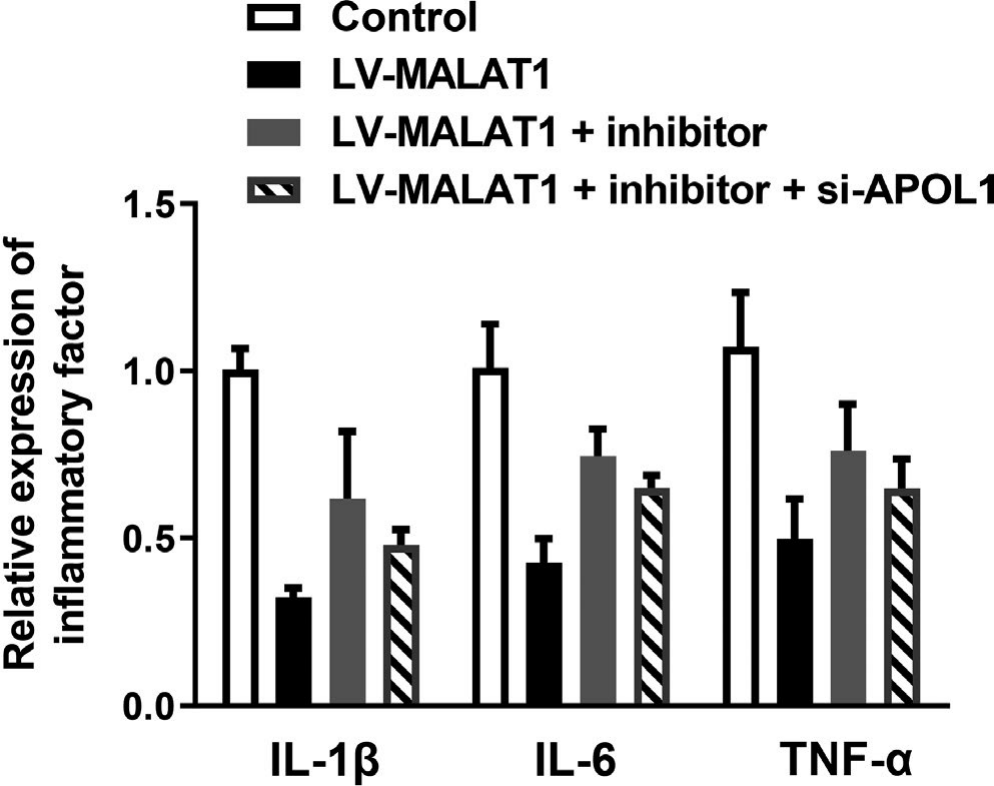
Effects of MALAT1/miR‐204/APOL1 axis on inflammation in hypoxia‐treated HK‐2 cells

Furthermore, as depicted in Figure 9A, after hypoxia conditions, the expression of APOL1 was gradually increased with the augment of treatment time of hypoxia. Then, the RT‐qPCR result in Figure 9B demonstrated the transfection efficiency of APOL1 was successful. Then, cell proliferation and apoptosis results in Figure 9C–F demonstrated that silencing of MALAT1 could increase hypoxia‐treated HK‐2 cell proliferation while decrease apoptosis; however, the variance could be partially counteracted by miR‐204 inhibitor. Compared with LV‐MALAT1 and miR‐204 inhibitor group, co‐transfection with si‐APOL1 could increase the proliferative capability while reduce the apoptotic ability of hypoxia‐induced HK‐2 cells. In addition, the result in Figure 10 validated that inhibition of MALAT1 could ameliorate inflammation response in HK‐2 cells after hypoxic treatment. Compared with LV‐MALAT1 group, co‐transfection with miR‐204 inhibitor could partially reverse the inflammation inhibited by MALAT1 silencing. Meanwhile, in comparison with LV‐MALAT1 + inhibitor group, co‐transfection with si‐APOL1 could aggravate the inflammation in HK‐2 cells after experiencing hypoxia.

## 4 DISCUSSION

In the present study, we investigated the biological roles of lncRNA MALAT1 and miR‐204 in AKI. In AKI patient serum and hypoxic HE‐2 cell models, MALAT1 expression was elevated, whereas miR‐204 expression was downregulated. MALAT1 knockdown reversed the effects of hypoxic treatment on HK‐2 cell proliferation, apoptosis, and inflammation through sponge‐mediated miR‐204. Furthermore, we observed that the NF‐κB pathway was inactivated in hypoxic HK‐2 cells, and miR‐204 inhibition abrogated the effect of MALAT1 on HK‐2 cells after hypoxic treatment by suppressing NF‐κB signaling.

Sepsis was a syndrome of the systemic inflammatory response caused by microbial infection.^20^ AKI, a common complication of sepsis, caused much attention due to its high mortality rate of 75% and multiple complications such as chronic kidney diseases.^21^ Some studies have reported that AKI was induced in more than 50% of patients with sepsis,^5^ leading to renal insufficiency and overproduction of inflammatory factors. It has been established that the pathogenesis of AKI involved multiple aspects, including renal inflammation, ischemia, acute hypoxia, oxidative stress, and microcirculatory disorders.^22^ However, the specific mechanisms behind AKI remained unclear. In recent years, an increasing number of complex interactions between lncRNAs and miRNAs were involved in the development of heterogeneous diseases, including AKI.^23^, ^24^ For example, lncRNA NEAT1 mediated hypoxia‐triggered apoptosis of renal tubular epithelial cells via sponging let‐7b‐5p.^25^ Silencing of lncRNA XIST targeted miR‐142‐5p/PDCD4 axis to ameliorate AKI development.^26^ In HK‐2 cells, lncRNA PVT1 promotes LPS‐induced septic AKI by regulating TNF‐α and JNK/NF‐κB.

Numerous studies have shown that lncRNAs can be stably presented in the blood through membrane vesicles such as exosomes and microvesicles, thus becoming promising biomarkers for disease diagnosis and prognosis.^27^, ^28^ Long non‐coding RNAs (lncRNAs) are a group of widely expressed non‐coding RNA molecules more than 200 nucleotides in length that regulate the expression of functional genes and play a key role in a variety of pathogenic conditions such as cardiovascular diseases,^29^ cancers,^30^ and AKI.^31^ Among them, lncRNA MALAT1 has recently been recognized as an essential regulator involved in cancer development,^32^ innate immunity,^33^ and viral infection.^34^ For example, lncRNA MALAT1 promoted hepatocellular carcinoma development by regulating SRSF1 and activating mTOR signaling.^35^ LncRNA MALAT1 could suppress IRF3‐initiated antiviral innate immunity by inhibition of TDP43.^33^ In addition, in renal cell carcinoma, MALAT1 can regulate the miR‐203/BIRC5 axis to accelerate tumor progression.^36^ Recently, it has been reported that lncRNA MALAT1 is an ideal marker for the diagnosis of sepsis.^37^, ^38^, ^39^ In the present study, we found that MALAT1 expression was elevated in AKI and functioned as an important diagnostic marker for AKI with high sensitivity and specificity. More importantly, knockdown of MALAT1 inhibited hypoxia‐induced apoptosis of HK‐2 cells and increased cell proliferation. These results suggested that MALAT1 can play a crucial role in the development of AKI as a biomarker for AKI therapy.

MicroRNAs (miRNAs) were a class of highly conserved non‐coding RNAs that played key roles in cell differentiation, metabolism, proliferation, and apoptosis.^40^, ^41^ In recent years, an increasing number of studies have shown that miRNAs were involved in the progression of sepsis.^42^, ^43^ Mechanistically, lncRNAs can act as ceRNAs to sponge miRNAs.^44^ Previous studies have revealed a prevalent interaction between lncRNA MALAT1 and miR‐204.^45^, ^46^, ^47^, ^48^, ^49^, ^50^ Combined with the bioinformatics tool, we predicted that miR‐204 was a target of lncRNA MALAT1. Consistent with previous studies, our results confirmed the targeted relationship between MALAT1 and miR‐204 in HK‐2 cells through luciferase reporter assay and RNA pull‐down assay. Recently, miR‐204 was considered to exert a reno‐protective effect in kidney diseases. For instance, Cheng et al.^51^ proposed that elevated expression of miR‐204 played a vital role in protecting against chronic renal injury through targeting the SHP2/STAT3 axis. Xiong et al.^52^ illustrated that miR‐204 was downregulated in renal cell carcinoma, inhibiting cancer development by preventing BAB22A expression. In ischemia‐reperfusion‐triggered AKI, miR‐204 was reported to protect against chronic fibrotic changes in renal tubes.^53^ In the present study, the results showed that miR‐204 expression was significantly suppressed in serum and hypoxia‐treated HK‐2 cells of AKI patients. As a target of lncRNA MALAT1, miR‐204 was negatively regulated by MALAT1, which could reverse the promotion of AKI progression by MALAT1.

APOL1 received increasing attention because of its important role in the pathophysiology of sepsis.^54^, ^55^ Recent studies have shown that APOL1 can interact with miRNAs, playing an important role in a variety of diseases.^56^, ^57^ In the present study, we uncovered that APOL1 was a target of miR‐204 and was negatively regulated by miR‐204. Western blot results showed that knockdown of lncRNA MALAT1 inhibited miR‐204 expression, while transfection of miR‐204 inhibitor reversed this effect. Finally, functional experiments elucidated that MALAT1 knockdown could enhance the proliferation while inhibit apoptosis in hypoxia‐induced HK‐2 cells. However, co‐transfection with miR‐204 inhibitor could partially abrogated the effects induced by MALAT1 silencing. Finally, compared with transfection with MALAT1 silencing and miR‐204 silencing, APOL1 knockdown could partially reverse the effects induced by miR‐204 inhibitor as well. The above results suggested that MALAT1 knockdown could regulate sepsis‐mediated AKI cell proliferation and apoptosis through the miR‐204/APOL1 axis.

Sepsis‐mediated AKI involves multiple factors, including endothelial injury and dysfunction, inflammation, and adaptive cellular responses to injury.^58^, ^59^, ^60^ A body of evidence suggested that the pathogenesis of sepsis‐induced AKI was thought to be caused by a pro‐inflammatory response.^61^, ^62^ Levels of cytokines such as IL‐1β, IL‐6, and TNF‐α have been shown to correlate with the severity and mortality of septic AKI.^63^, ^64^ The present study showed that the secretion of pro‐inflammatory cytokines IL‐1β, IL‐6, and TNF‐α was significantly increased in HK‐2 cells after hypoxic treatment. More importantly, lncRNA MALAT1 silencing reduced the levels of IL‐1β, IL‐6, and TNF‐α in HK‐2 cells after hypoxia treatment, while miR‐204 inhibitor had a pro‐inflammatory effect on inflammation.

The NF‐κB signaling pathway has been shown to be involved in the regulation of a variety of biological processes, including AKI.^65^, ^66^ It has been shown that activation of NF‐κB reduced inflammation while mitigating sepsis‐induced organ damage.^67^ LncRNAs NEAT1 and PVT1 were found to affect LPS‐triggered septic AKI by regulating the NF‐κB pathway by Chen et al.^68^ and Huang et al.^69^ Therefore, we hypothesized that NF‐κB activation may be involved in protecting sepsis‐triggered AKI. Compared with the Chen's study,^68^ our study further validated a downstream target of miR‐204 and a upstream target of NF‐κB pathway, APOL1, which participated in regulating hypoxia‐induced HK‐2 cell proliferation, apoptosis, and inflammation. In a word, in our study, we found that MALAT1 knockdown activated NF‐κB activation, while miR‐204 inhibition significantly counteracted this effect.

In some aspects, our experiments still have some limitations. A larger number of clinical samples is important in further investigations. Meanwhile, besides p65, there are other essential factors in NF‐κB signaling pathway, such as p50. In the future, we will further study the effects of lncRNA MALAT1/miR‐204/APOL1 axis on p50. Moreover, the expression of inflammatory factors in vivo caused by hypoxic damage was very complex. The dynamic balance of various inflammatory factors including pro‐inflammatory factors and anti‐inflammatory factors was destroyed, which was more crucial than the change of single inflammatory factor. Thereby, we will examine the dynamic changes between anti‐inflammatory and pro‐inflammatory factors induced by hypoxic injury. Finally, to further elucidate the role of lncRNA MALAT1 in AKI mice, in vivo experiments are needed in the future.

Taken together, our study reveals that lncRNA MALAT1 can sponge miR‐204 to regulate HK‐2 cell proliferation, apoptosis, and inflammation after hypoxia treatment. More importantly, knockdown of lncRNA MALAT1/miR‐204/APOL1/NF‐κB axis may be a potential therapeutic target to contribute to the treatment of AKI.

## CONFLICT OF INTEREST

None.

## Data Availability

The datasets used and/or analyzed during the current study are available from the corresponding author on reasonable request.
